# Reconstructing Source-Sink Dynamics in a Population with a Pelagic Dispersal Phase

**DOI:** 10.1371/journal.pone.0095316

**Published:** 2014-05-16

**Authors:** Kun Chen, Lorenzo Ciannelli, Mary Beth Decker, Carol Ladd, Wei Cheng, Ziqian Zhou, Kung-Sik Chan

**Affiliations:** 1 Department of Statistics, University of Connecticut, Storrs, Connecticut, United States of America; 2 College of Earth, Ocean and Atmospheric Sciences, Oregon State University, Corvallis, Oregon, United States of America; 3 Department of Ecology and Evolutionary Biology, Yale University, New Haven, Connecticut, United States of America; 4 PMEL, NOAA, Seattle, Washington, United States of America; 5 Joint Institute for the Study of the Atmosphere and Ocean (JISAO), University of Washington, Seattle, Washington, United States of America; 6 Department of Statistics and Actuarial Science University of Iowa, Iowa City, Iowa, United States of America; North Carolina State University, United States of America

## Abstract

For many organisms, the reconstruction of source-sink dynamics is hampered by limited knowledge of the spatial assemblage of either the source or sink components or lack of information on the strength of the linkage for any source-sink pair. In the case of marine species with a pelagic dispersal phase, these problems may be mitigated through the use of particle drift simulations based on an ocean circulation model. However, when simulated particle trajectories do not intersect sampling sites, the corroboration of model drift simulations with field data is hampered. Here, we apply a new statistical approach for reconstructing source-sink dynamics that overcomes the aforementioned problems. Our research is motivated by the need for understanding observed changes in jellyfish distributions in the eastern Bering Sea since 1990. By contrasting the source-sink dynamics reconstructed with data from the pre-1990 period with that from the post-1990 period, it appears that changes in jellyfish distribution resulted from the combined effects of higher jellyfish productivity and longer dispersal of jellyfish resulting from a shift in the ocean circulation starting in 1991. A sensitivity analysis suggests that the source-sink reconstruction is robust to typical systematic and random errors in the ocean circulation model driving the particle drift simulations. The jellyfish analysis illustrates that new insights can be gained by studying structural changes in source-sink dynamics. The proposed approach is applicable for the spatial source-sink reconstruction of other species and even abiotic processes, such as sediment transport.

## Introduction

Source-sink dynamics are very common ecological processes that affect species abundance and distribution in spatially structured landscapes [1]. Source-sink dynamics arise due to the heterogeneous pattern of productivity of patches across the landscape, where the most productive patches function as sources of propagules for the less productive patches. This established population-dynamics definition of sources and sinks emphasizes the effect of habitat quality on demographic rates [2]. The ensuing dynamics can give rise to several complex population systems, ranging from tightly linked source-sink population to less connected metapopulation [3] or sympatric complexes (genetically structured populations that share portions of their habitats during part of their life cycle).

Sources and sinks can also pertain to origins and destinations of dispersive stages [4]–[6]. Tightly linked source-sink dynamics may occur through ontogeny, where organisms move through different habitats during the course of their life-cycle. Ontogenetic shifts of spatial distribution are particularly common in marine systems, where many species have a pelagic larval phase which drifts with the prevailing currents, before settling in nursery locations [7]. In such cases, the spawning locations of species with a long pelagic larval duration can be represented as sources, and the settling locations of the juvenile or adult stages as sinks. Here, we employ this definition of source-sink dynamics i.e., origins and destinations of dispersive stages to the dispersal of scyphozoan jellyfish in the eastern Bering Sea (see below). Source-sink dynamics are common in many tropical marine (e.g., [8]) and freshwater (e.g., [9]) systems, giving wider applicability to our research. In addition to the source and sink patches, another feature that these systems share is the presence of a directional flow that moves particles between the two sites. Directional flow may operate in a variety of systems; for example, ocean currents carrying larvae, winds carrying plant seeds, or migratory pathways carrying pathogens with migrant animals. Population connectivity and directionality of flow between sources and sinks have important applications for the management and conservation of many species, in particular, the design of protected (or management) areas [10]–[12].

Usually we only have partial knowledge of the source-sink spatial assemblage, limiting our understanding of the temporal dynamics of the source-sink systems [13]. Limited information about spawning locations can inhibit understanding of how recruitment variability in the adult stock is affected by changes in currents and larval supply from source locations [14]. Analogously, for spreading of pathogens, we may be aware of focal points in which the diseases occur but we do not know the origins, such as the hosts in which it is sustained [15].

In principle, the spatial assemblage of the source-sink locations can be reconstructed in its entirety if we have knowledge of one of the two components of the source-sink system and of the mechanisms by which particles are carried from one site to the other (e.g., currents, winds, migration pathways). Typically, the process of reconstructing the spatial assemblage of source-sink systems involves the simulation of propagules drifting from the putative source to the putative sink locations, and the comparison of the model outcome with the field observations. In marine ecology this practice is becoming increasingly common, with the development of high resolution ocean circulation models coupled to individual based models, which simulate ocean currents and individual behavior during the drifting phase [16]. There are however unresolved challenges associated with the interpretation of output from these coupled models. Namely, how do we statistically compare the output of a drift simulation with the knowledge that we have gathered from field observations? And how do we make ecological inferences from such comparisons? Meeting these challenges requires a new framework that addresses the following critical issues: (i) simulated propagules rarely coincide in space with field observations even though they may be close in proximity, (ii) potential sources may be of uneven productivity, with linkages between potential sources and sinks being of variable strength and sparse, i.e., many of them may be unlinked, and (iii) the source-sink dynamics may undergo temporal, structural changes. Here we propose a new statistical method to overcome these challenges, which we apply to spatially define the source timing and locations of scyphozoan jellyfish in the eastern Bering Sea (EBS).

Most scyphozoan jellyfish have a complex, two-part life cycle. The pelagic, sexually-reproducing medusa phase is the most conspicuous part of the life cycle. However, the medusae are propagated asexually from a perennial benthic stage called a polyp (i.e., scyphistoma; [17]), which are the source of medusae. The release of the immature medusae (ephyrae) from polyps is known as strobilation (i.e., disk formation by transverse fission). The polyps develop from sexually-produced larvae, which are released from the medusae then settle onto substrate. The polyps are small (a few mm in length), typically hang underneath rocks and shells [18], [19] and thus, they are notoriously difficult to find in the field. The source locations of jellyfish (i.e., the polyp colonies) from which most “blooming” species develop have remained by-and-large a mystery [20]. Since medusa population sizes reflect the reproductive success of the polyp stage [21], lack of knowledge about the source populations have limited the understanding of the drivers of coastal jellyfish blooms [20].

A northwestward expansion of jellyfish distribution occurred in the EBS after 1990 [22], [23]; see Fig. S1. Mechanisms underlying this shift in distribution are unknown, although several hypotheses have been postulated: (i) stronger oceanic transport from the southeastern to the northwestern part of the survey area carried more jellyfish to the northwest after 1990, (ii) warmer temperatures after 1990 allowed increased jellyfish production or allowed polyps to proliferate in the northwestern EBS, and (iii) oceanic transport and changing production interact to result in the shift in jellyfish distribution, see [23], [24].

Here, we evaluate hypothesis (iii), that changes in both the circulation and jellyfish productivity interact to result in the change in jellyfish distribution. We do this by (i) applying a new model for the reconstruction of sources and sinks and (ii) fitting the model to the spatial distribution of jellyfish abundance based on summer bottom trawl survey data and oceanographic circulation model output, for the periods before and after the observed shift in jellyfish distribution, i.e., 1982 to 1990 and 1991 to 2004.

The proposed method is generally applicable for source-sink reconstruction to study ecological processes such as, the range expansion of introduced species facilitated by ocean currents [25], the emergence of pathogens from “reservoir” wildlife species [15], the long-distance aerial dispersal of plant pathogens [26], and source-sink analysis in abiotic systems, e.g. linking sediment dispersal to associated stratigraphy [27].

## Materials and Methods

### Jellyfish Biomass and Study Region

Jellyfish biomass and distribution for the period 1982–2004 were obtained from quantitative bottom trawl surveys of groundfish on the EBS shelf conducted by the Alaska Fisheries Science Center (AFSC) [22], [28], [29], the data of which can be downloaded from the RACE groundfish data base http://www.afsc.noaa.gov/RACE/groundfish/survey_data/data.htm.

**Figure 1:**
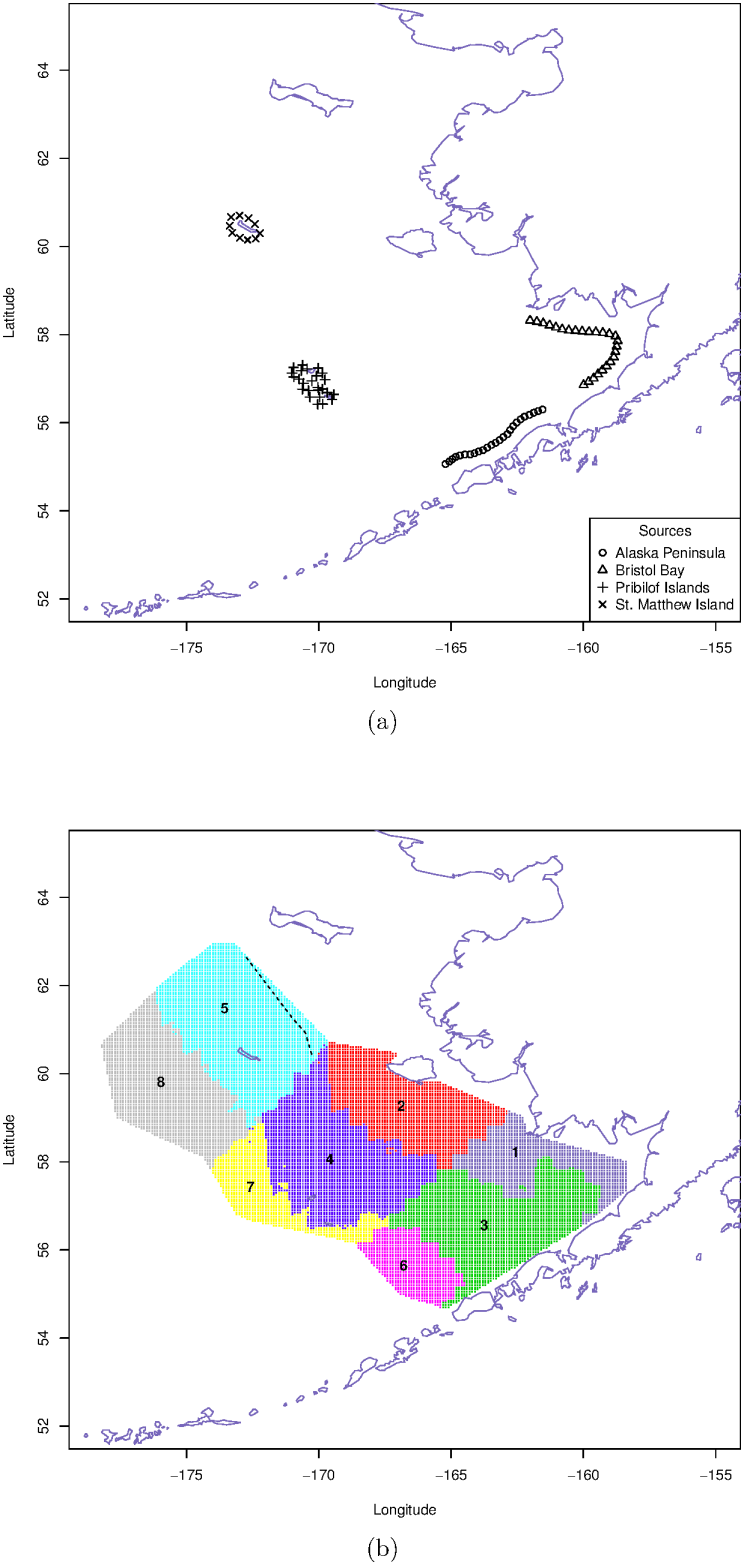
Map of the eastern Bering Sea, indicating 4 sources in the left panel and 8 sink regions on the right panel.

**Figure 2:**
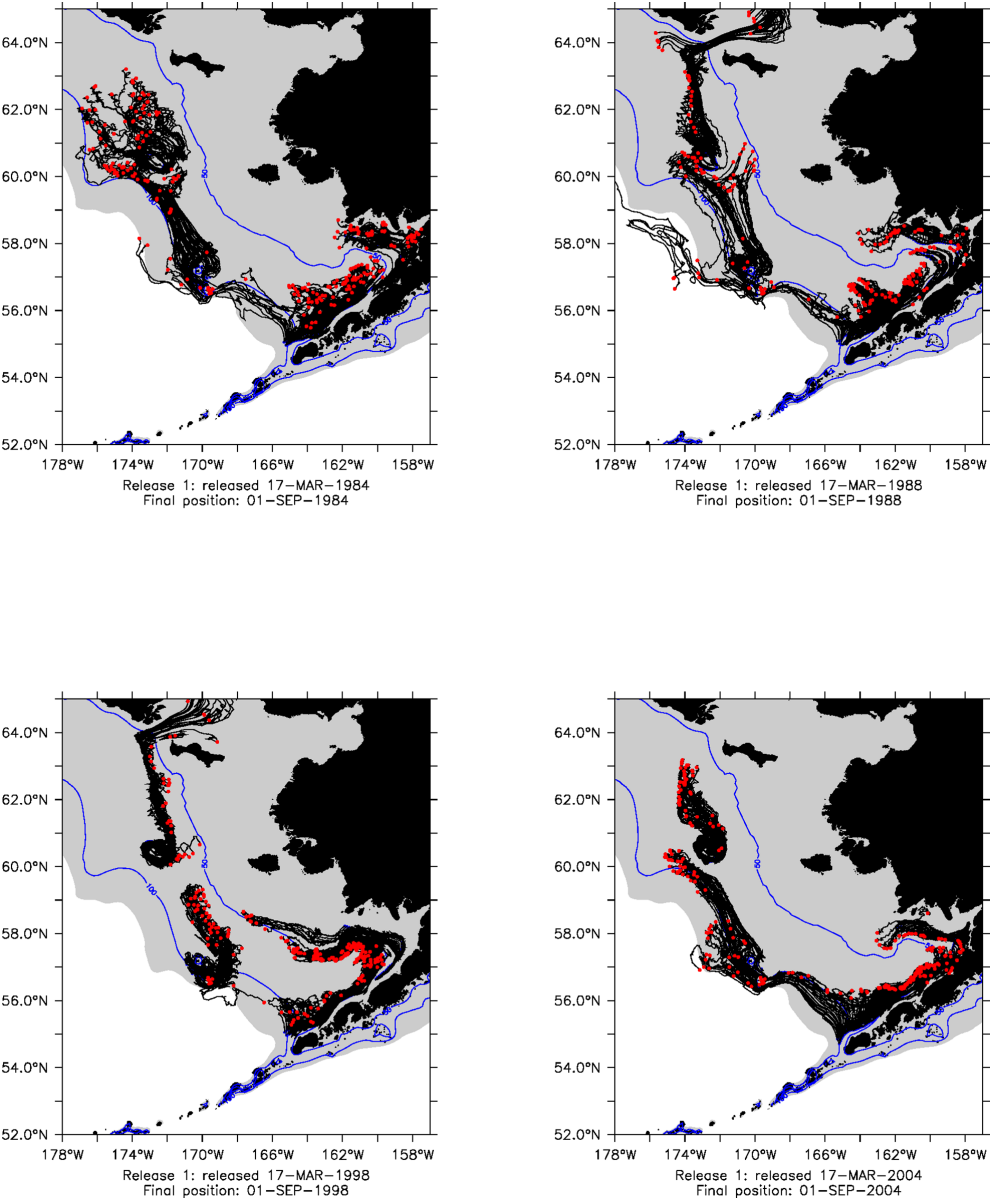
Black lines show the trajectory and red dots final location of propagules released in the first release in 1984, 1988, 1998 and 2004. Shelf (shallower than 200 m) is denoted by gray shading. 50 m and 100 m isobaths shown as blue contours.

During the surveys, trawls were deployed at 356 stations arranged in a grid pattern (36 km×36 km) during daylight hours from late May through August. Station depths ranged from 15 m in the southeast corner of survey area to nearly 200 m along the shelf break on the western edge of survey area (Figures 1, S2). The trawl, with a 26.5 m headrope and 34.1 m footrope with graded mesh (10 cm at the mouth to 3.8 cm in the codend), was towed on the bottom for 30 min at 5.4 km per h [30]. When fishing on the bottom, the net height was approximately 2.5 m and the trawl remained open and fished throughout the period of deployment and recovery. Catches of all large jellyfish (bell diameters 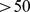 mm), primarily *Chrysaora melanaster* [31], were weighed at sea and standardized to catch per unit effort (CPUE in kg
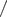
ha, where 1 ha = 10,000 m^2^) (see [28], for details). Since jellyfish are distributed throughout the water column (30–40 m mean depth; [29], [32]), the medusa biomass estimated from the bottom trawl catch used here are considered an index of relative abundance that is comparable among stations and years. Although jellyfish data extend to the present, the circulation model output (described below) was only available until 2004, which limited the time frame of our analysis. Total summer jellyfish CPUE for each year were estimated for each of the 8 sink regions described below (and shown in Fig. 2b), and are logarithmically transformed after adding 1, for variance stabilization (see *Model Fitting and Diagnostics* in *File S1*).

### Circulation Model

To examine the influence of ocean circulation on the distribution and biomass of jellyfish, we used the Regional Ocean Modeling System (ROMS) to simulate jellyfish dispersals in the ocean. ROMS is a free-surface, terrain-following, primitive equation ocean circulation model driven by atmospheric forcing, and has been used extensively in the oceanographic community. An overview of ROMS and its numerical methods can be found in [33], [34] and references therein. The ROMS used in this study covers the entire Northeast Pacific (NEP) including the Bering Sea with 10 km horizontal resolution and 43 vertical layers. Validation of ROMS NEP simulations is discussed in [35]. Studies of vertical distributions indicate that EBS jellyfish cluster at approximately 30–40 m depth [29], [36] and scyphozoan ephyrae were found in the upper 40 m (Fig. S4). To model jellyfish advection, propagules were introduced at 3 depths in the model (20, 30, and 40 meters) from four source regions on the EBS shelf: the north side of the Alaska Peninsula, Bristol Bay, the Pribilof Islands, and St. Matthew Island (Fig. 2). These regions were determined by analysis of Bering Sea bottom types (National Oceanic and Atmospheric Administration 2010, Fig. S5) to be hard and rocky and therefore, potential jellyfish polyp habitat. Immature scyphomedusae (i.e., ephyrae; see Fig. S3) have been observed near the Alaska Peninsula source location (Fig. 1) With the exception of the Alaska Peninsula region (100 locations), each of the source regions included 50 individual source locations for a total of 250 individual source latitude/longitude locations (Fig. 1a). The propagules were first released on March 15th of each year, with additional releases every other day until June 5th (40 releases), see Fig. 2. The first releases occurred 2 months prior to the observation of ephyrae in limited plankton sampling ([31]; Fig. S4). Propagule releases continued until early June, since medusae released after this time are not likely to be collected during AFSC groundfish survey.

Mean circulation on the southeastern Bering Sea shelf is typically weak (generally 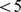 cm s^−1^) and northwestward from late spring (typically May) through mid-autumn. Flow is stronger and more organized along frontal zones located along the 100 m and 50 m isobaths. During the winter, the frontal structures break down and flow is less organized [37]. We chose March 15 as the initial propagule release date so that propagule releases span the winter to spring transition in every year. Altogether, a total of 30,000 propagules (250 locations
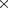
3 depths
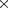
40 releases) were released into the model ocean each year. Once released, their depths were kept constant and their horizontal trajectories were simulated using ROMS internal particle-tracking algorithm. The ROMS integrations were carried out from March 15th to September 30th of each year (1982 to 2004), and daily propagule locations were recorded during model integrations. These data are used to derive a set of covariates, denoted as 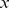
’s below, needed for source-sink reconstruction, which may be downloaded at a link in the *File S1*.

Ocean circulation models will not exactly reproduce the circulation of the real ocean due to errors in surface forcing and model physics, and finite model resolution. By comparing ROMS simulated ocean velocities with observed velocities derived from satellite tracked drifters, we found that the ROMS simulation captures the observed current directions reasonably well, but the simulation tends to underestimate the current amplitude. This underestimation is typical of coarse resolution ocean circulation models. To investigate the effects of such model limitations on the source-sink reconstruction, we have performed some sensitivity experiments to be discussed below.

Since trawl survey locations seldom overlap with the trajectories of particles simulated based on the circulation model described above, we use spatial aggregation to facilitate comparing circulation model outputs with field data. The EBS is naturally divided in the east-west direction into the inner, middle and outer domains, along the 50 m and 100 m isobaths [38]. In studying shifts in the jellyfish distribution in the EBS, [23] suggested dividing the EBS in the north-south direction into three regions, with two straight line boundaries running from the point with latitude and longitude (58°N, 174.1°W) to the point (60.7°N, 169.4°W), and another from (55.7°N, 169.2°W) to (59.1°N, 163°W). These regions delineate 8 sinks (the northernmost inner shelf region is neglected due to scarcity of data, see Fig. 1).

### A Sparse Multi-component Multivariate Regression Model for Source-Sink Reconstruction

We propose a method for source-sink reconstruction for the scyphozoan jellyfish system, where the near-shore polyp colonies are the sources and the offshore waters that the medusae occupy are sinks; mathematical details and derivations of some theoretical properties of the method are given in [39]. However, this method is generally applicable to various biological systems where the propagules are passively dispersed from several sources to sinks. Suppose there are 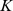 potential sources of propagules that are dispersed to the sink which is subdivided into 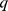 regions over each of which the annual average density of the species in year 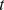 equals 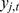, where 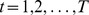 and 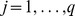. We consider the case that no observational data are available on the propagules, which is the case for jellyfish in this system; small medusae 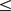
50 mm are not sampled by the bottom trawl survey and the immature medusae (i.e., ephyrae, 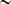
2 mm in diameter) are rarely found in routine plankton sampling. Likewise, the polyps from which the ephyrae are released are also very small (a few mm in length) and are difficult to find in the field [20].

Suppose that at source 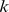, 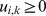 equals the probability that a propagule is released on day 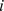 since a fixed date in a year, say March 15, for 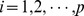. In order to do the source-sink reconstruction, i.e., to estimate which source contributes to which part of the sink, we need information concerning the relative strength of passive transport from any particular source to any particular region of the sink. For the jellyfish system in the EBS, ephyrae and small medusae are assumed to be passively transported by ocean currents. As detailed in the data section, based on particle drift according to an ocean circulation model, we can estimate the fraction of time a (random) immature medusa from source 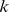 that was released on day 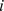 of year 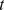 visited region 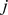 before the end date of the annual survey; let this fraction be denoted by 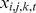. (More generally, 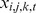 denotes the fraction of time that a propagule, released at source 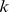 on day 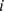 of year 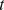, visited region 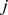 in year 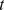.) Ignoring survival, the maximum contribution from source 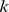 to each region 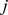 in year 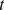 is equal to 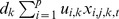, where each 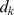 is a source-specific multiplier related to the overall productivity of source 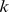 (A potential source is not a true source if its 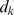 equals 0.) The propagules (i.e., jellyfish ephyrae) from each source (polyp colony) are advected to various regions. In practice, to calculate the contribution from a particular source to each region, the preceding formula has to be modified to account for differential advection and survival rates between the source and the regions in the sink. Let 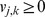 denote the (relative) probability that a propagule survived and drifted from source 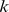 to region 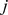. Hence, in year 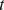, the density of the species in region 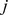 is composed of contributions from all the sources and is expected to equal 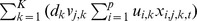, on average. The preceding considerations lead to the following model for source-sink reconstruction:
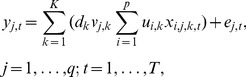
(1)where 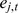 are pairwise uncorrelated error terms of zero mean and constant variance. Also, see [39] for further technical details. In summary, the 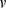
’s in the proposed model are the survival probabilities for a propagule per each source-sink trip, averaged over the study period. The sums of the 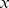
’s weighted by the 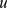
’s, the propagule release time probabilities, serve as proxies for the maximal contribution from a source to a sink per arbitrarily fixed production level, assuming no mortality. Thus, the model tries to use the information in the 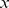
’s and 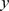
’s to tease out (i) the source-sink linkage pattern, i.e., which 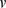
’s are non-zero, (ii) the source-specific production level, i.e., the 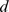
’s in Eqn. (1), and (iii) the source-specific larva release time curves.

Note that the 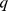 sink regions define a spatial discretization of the sink, providing an effective solution to the problem that simulated propagules rarely intersect field observations spatially. The parameter 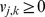 will be referred to as the “sink effect” from source 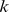 to region 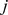. For 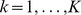, let 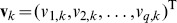 which is generally a *sparse* and non-negative vector, i.e. most components are zero because the propagules in any particular source are expected to drift into only a few nearby sink regions. (Here, the superscript 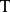 stands for the transpose of the vector.) The parameter 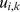 is proportional to the probability that a propagule is released on day 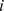 at source 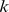. For source 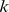, the vector 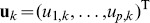 gives the release time curve (the probability mass function of the timing of propagule release) which is assumed to be a smooth and non-negative function of time. The smoothness assumption is enforced by expressing 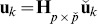 with 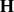 consisting of cubic spline basis functions of degrees of freedom 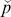, and requiring 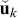 to be sparse to achieve smoothness. Because 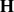 can be absorbed into the covariate 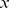
’s, without loss of generality, we shall focus on the requirement that 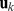 is a sparse vector, for each 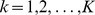.

The proposed model connects the observed spatial distribution of jellyfish in various regions of the EBS (sinks) with the unobserved ephyrae release from a few potential polyp colonies (sources). The model can be used to infer the (net) productivity of the potential sources, i.e. the average annual (net) density of the propagules at source 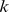 can be calculated as
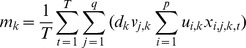
For the model to be identifiable and interpretable, we require that for a fixed 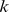, where 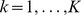, all components of 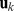 are non-negative and they sum to 1; similarly all components of 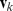 are non-negative and they sum to 1. Without these constraints, the model parameters are not fully identifiable because we can multiply the 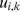
’s by some non-zero constant and divide the 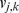
’s by the same constant.

The source-sink reconstruction model can be estimated by the method of penalized constrained least squares, i.e. minimizing the sum of squared errors plus a penalty term for each pair of non-negative constrained parameter vectors 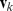 and 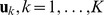 that is proportional to the magnitude of the weighted outer product of the pair of vectors [40], specifically, by minimizing the following objective function:
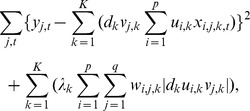
where for any fixed 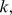 the non-negative parameters 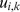
’s and 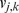
’s are subject to the constraints that they sum up to 1, 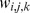
’s are some pre-determined data-driven weights and 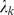
’s are non-negative tuning or regularization parameters; see [39]. Our regularization method is analogous to the adaptive Lasso method [41] in ordinary linear regression, and the weighted penalization scheme is designed to prompt model estimation and selection consistency [40]. Setting all tuning parameters to 0 and ignoring the nonnegativity constraint yields the method of least squares which, however, generally results in non-sparse, non-smooth and uninterpretable source-sink reconstruction, assuming that there are adequate data for estimation. On the other hand, setting the tuning parameters to extreme large values will force all the parameter estimates to become zero. Thus, it is pivotal to choose appropriate tuning parameters based on the data. Cross-validation and some information criterion, e.g. the Akaike information criterion (AIC) [42], may be used to choose the tuning parameters. (We used AIC for determining the tuning parameters in all results reported below.) With the tuning parameters estimated from the data, the penalized least squares method attempts to find a model that fits the data well, yet with sparse sink effects and smooth release time curves; see [39]. [43] has recently shown that the residual bootstrap can be used to consistently estimate the distribution of the adaptive Lasso estimators. We thus follow [43] and use a residual bootstrap method to access the uncertainty in parameter estimation, see *Bootstrap* in *File S1*.

Biological systems often undergo structural changes in their source-sink dynamics, e.g., triggered by the onset of a new climate regime that alters the relative connectivity strength between the source-sink pairs. Consider the problem of testing the hypothesis 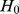 that a biological system undergoes a change in its source-sink dynamics in year 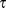, i.e. the source-sink dynamics in the period ending in year 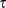 differs from that of the period after year 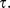 The parameter 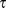 may be inferred from other information. The spatial distribution of jellyfish in the EBS has been shown to have shifted after 1990 [23], so it is of interest to test whether or not the source-sink jellyfish dynamics underwent a structural change in 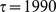. This can be done using a permutation test as follows: Under the null hypothesis of no change in the source-sink dynamics, we can shuffle the data cases by permuting the year. For each permutated dataset, we refit the model to the two separate periods (pre- and post-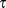) and compute a test statistic defined as the sum of the AIC of the model using data from the “pre-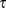
” period and the AIC of that from the “post-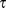
” period; denote the test statistic by 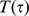. Repeat this procedure B, say 500, times, resulting in B permutated test values. The p-value of the null hypothesis of no structural change is then asymptotically equal to the fraction of the permutation test values greater than the observed test value. For the case of unknown 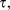 it can be estimated by the year which minimizes 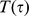 subject to the constraint that there are sufficient data in both pre- and post-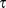 periods.

### Sensitivity Analysis

To understand the effects of the systematic underestimation in the modeled ocean current, we created several sets of “shifted” ROM outputs based on the original ROMS simulation, by shifting the positions of the propagules at any given time towards their future counterparts. Denote the rate of shifting by 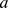, e.g., 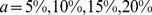. For any propagule released on day 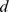, its longitude and latitude at day 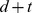 are set to be the ROMS-simulated values on day 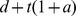 when this number is an integer. Otherwise, the longitude and latitude values are obtained by interpolation based on the ROMS-simulated positions on the two integer days that are closest to 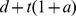. In this way, the propagule positions are shifted forward to counter the systematic underestimation of the current velocities, and the effect of shifting is allowed to accumulate over time linearly. Each shifted ROMS dataset can then be used to compute the covariates 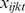, with which the source-sink reconstruction model is refit for assessing the effects of increasing 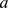.

Effects of random errors in the trajectory of the propagules on the source-sink reconstruction can be assessed by perturbing each 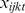 by randomly adding or subtracting 5% of its original value, and then refitting the model with the perturbed covariates. This process can then be repeated multiple times for assessing the sensitivity of the model fit to random errors.

## Results

The process underlying the jellyfish distributional shift into the north-western corner of the survey area in the EBS around 1990 [22], [23] may be revealed by contrasting the jellyfish source-sink dynamics over the period prior to and including 1990 (pre-1990 period) from that over the period starting on 1991 (post-1990 period). We fit the source-sink model defined by eqn. (1) separately to the data from the pre-1990 period and those from the post-1990 period. Model diagnostics (see *Model Fitting and Diagnostics* in *File S1*, Figures S6 and S7) suggest that the two-period model fit the data well. Also, there is strong evidence of structural changes in the jellyfish source-sink dynamics pre- and post-1990 (p-value of no change is less than 0.002), based on the permutation test with 500 replications (see *Model Fitting and Diagnostics* in *File S1*).

Table 1 reports the estimates of the sink effects 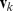 and the annual jellyfish production rate 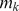 for each of the four potential sources, based on the model fitted to the pre-1990 data. The uncertainty in the parametric estimates are assessed by bootstrap, based on 400 replications. In particular, Table 1 reports the bootstrap probability that a coefficient equals 0 and the 90% confidence intervals of the coefficients. For some source-sink combinations, the ocean circulation model indicates zero time spent by the propagules during the study period. For such source-sink combinations, the sink effect parameters 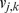 are then fixed to be zero, represented by asterisks in Tables 1 and 2. The 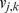 estimates are constrained to sum to 1 for a fixed 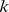 and therefore can be interpreted as the conditional probability that an ephyra released in source 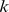 drifted alive to sink 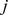. The sinks are labeled from 1 to 8, which are color-coded in Fig. 1. For the pre-1990 period, the estimates of the 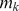
’s suggest that the Alaska Peninsula was the primary source and the Pribilof Islands the secondary source, while Bristol Bay and St. Matthew Island were not significant sources (bootstrap p-values of 0.14 and 0.33, respectively). Simulated jellyfish ephyrae released along the coastline of the Alaska Peninsula were advected into sinks 3 and 6, both adjacent to the Peninsula. Ephyrae released around the Pribilof Islands drifted into sinks 4 and 7 adjacent to the Pribilof Islands. Jellyfish initialized in Bristol Bay and around St. Matthew Island tended to drift to the sinks adjacent to them, specifically sinks 1 and 5 respectively, although they were not significant sources.

**Table 1:** Source-sink reconstruction (1982–1990).

	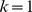 : AP			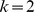 : BB			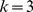 : PI			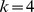 : SM		
	Est.	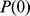	90% C.I.	Est.	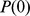	90% C.I.	Est.	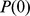	90% C.I.	Est.	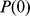	90% C.I.
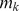	**1.65**	0 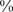	(1.18,1.97)	0.16	14 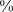	[0.00,0.54)	**0.50**	3 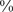	(0.13,0.71)	0.16	33 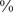	[0.00,0.25)
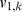	0.00	86 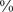	[0.00,0.14)	1.00	20 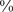	[0.00,1.00)	*		*	*	*	*
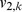	*	*	*	0.00	100 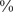	[0.00,0.00]	0.00	100 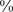	[0.00,0.00]	*	*	*
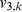	**0.07**	1%	(0.01,0.10)	0.00	61%	[0.00,1.00)	0.00	100 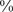	[0.00,0.00]	*	*	*
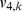	0.00	77 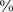	[0.00,0.21)	*	*	*	**0.08**	5 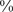	(0.0003,1.00)	*	*	*
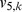	0.00	100 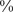	[0.00,0.00]	*	*	*	0.00	81 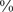	[0.00,0.79)	1.00	34 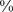	[0.00,1.00)
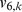	**0.93**	0 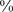	(0.29,0.96)	*	*	*	0.00	100 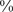	[0.00,0.00]	*	*	*
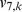	0.00	56.5 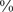	[0.00,0.41)	*	*	*	0.92	36 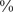	[0.00,0.94)	*	*	*
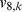	0.00	96 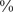	[0.00,0.00]	*	*	*	0.00	87 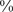	[0.00,0.84)	0.00	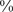 93	[0.00,0.93)

AP: Alaska Peninsula; BB: Bristol Bay; PI: Pribilof Islands; SI: St. Matthew Island. Est.: parameter estimate; 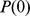: probability of being zero based on bootstrap; 90% C.I.: 90% confidence interval based on bootstrap (400 replications); when 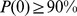, the C.I. is set to 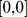, otherwise the end points of the C.I. are chosen as the 5th and 95th percentile of the bootstrap distribution. The underlined numbers are nonzero estimates, among which the significant parameters (at 10% significance level) are indicated in bold font.

**Table 2:** Source-sink reconstruction (1991–2004).

	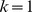 : AP			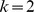 : BB			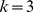 : PI			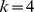 : SM		
	Est.	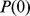	90% C.I.	Est.	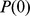	90% C.I.	Est.	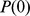	90% C.I.	Est.	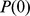	90% C.I.
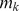	**2.76**	0 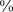	(2.09,3.29)	**0.62**	0 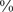	(0.33,1.08)	**1.78**	0 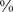	(1.16,2.24)	**1.71**	0 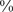	(1.29,2.06)
												
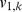	0.00	91 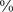	[0.00,0.36)	**1.00**	0 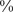	(0.06,1.00)	*		*	*	*	*
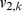	*	*	*	0.00	95 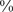	[0.00,0.72)	0.00	100 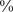	[0.00,0.00]	*	*	*
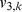	**0.11**	0%	(0.002,0.21)	0.00	81%	[0.00,0.87)	0.00	99 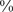	[0.00,0.00]	*	*	*
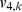	0.40	41 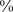	[0.00,0.94)	*	*	*	**0.01**	0 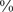	(0.002,0.03)	*	*	*
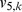	0.00	95 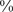	[0.00,0.52)	*	*	*	0.01	46 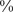	[0.00,0.04)	**0.01**	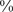 0	(0.002,0.05)
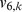	**0.49**	0 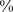	(0.01,0.88)	*	*	*	0.00	90 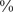	[0.00,0.32)	*	*	*
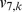	0.00	94 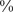	[0.00,0.03)	*	*	*	**0.05**	1 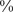	(0.02,0.17)	*	*	*
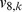	0.00	91 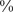	[0.00,0.77)	*	*	*	**0.93**	2 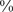	(0.55,0.97)	**0.99**	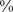 2	(0.95,1.00)

All the settings are the same as in Table 1.

For the post-1990 period, Table 2 suggests that jellyfish production experienced universal increases in all 4 sources. The coastline of the Alaska Peninsula remained the most productive source. There was a large increase in jellyfish production around the Pribilof Islands (the 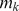 estimate increased from 0.50 to 1.78). At the same time, the coastline around St. Matthew Island and Bristol Bay became significant, productive sources (increasing from 0.16 to 1.71 for St. Matthew and from 0.16 to 0.62 for Bristol Bay). Moreover, there was an expansion in the drifting from the three significant sources to the sinks. After 1990, medusae originating along the Alaska Peninsula drifted into sink 4 (with better than even odds), in addition to sinks 3 and 6; those released around the Pribilof Islands were advected into sinks 5 and 8 (with better than even odds), beyond sinks 4 and 7; and jellyfish initiated along the St. Matthew Island drifted into sinks 8 and 5.

**Figure 3:**
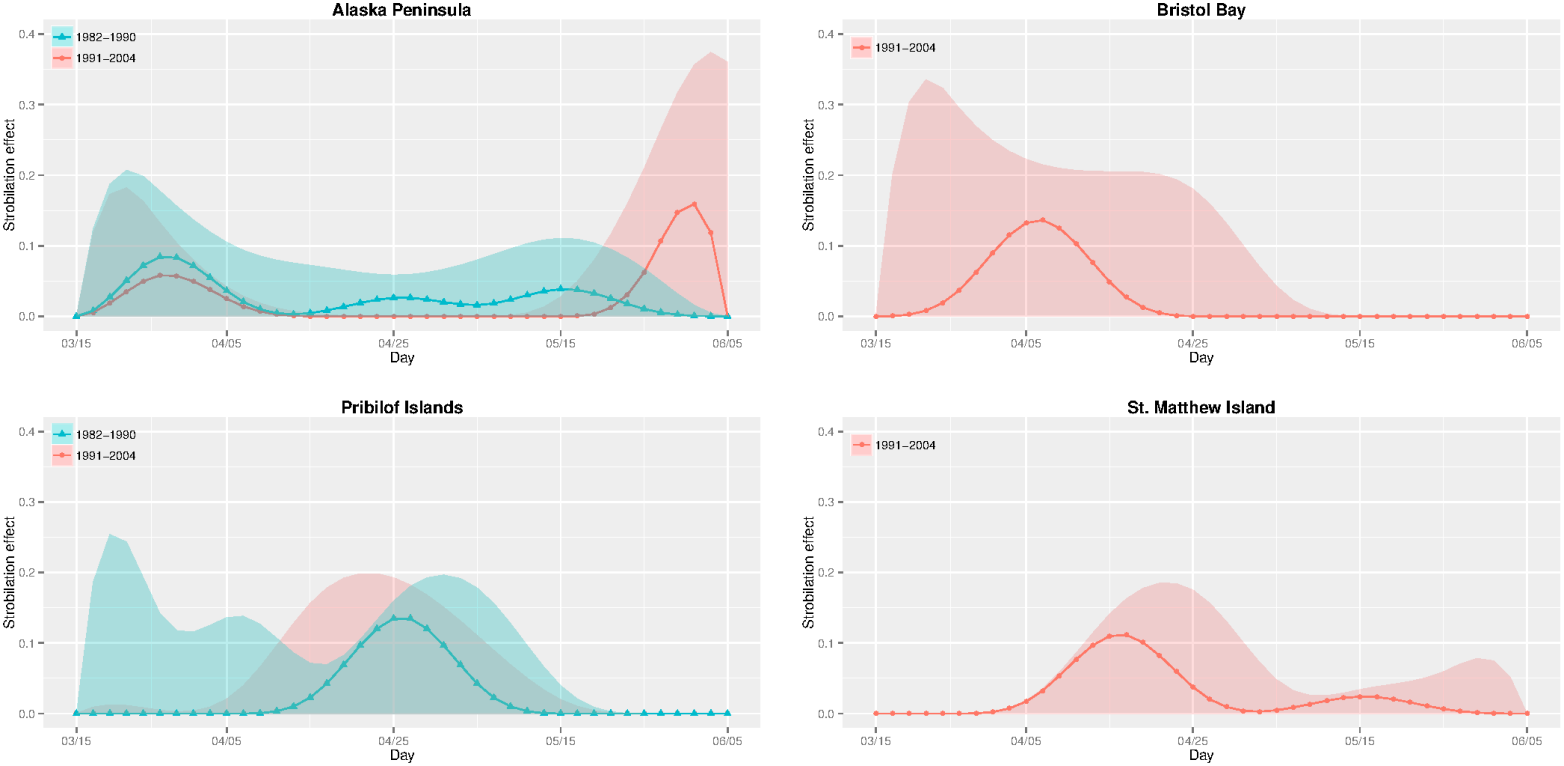
Release time (strobilation) curves (the estimated 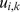
’s) for the periods 1982–1990 (blue triangles) vs. 1991–2004 (red circles) and their 90% point-wise confidence regions shaded blue (red) over the first (second) period. The estimated strobilation curves for the Pribilof Islands are almost identical for the two periods. The 80-day strobilation window is from March 15th to June 5th each year.

Fig. 3 plots the estimated probability mass functions of release timing of ephyra (i.e., strobilation) for the significant sources in the pre-1990 and the post-1990 periods. The discrete dots of the estimated probability masses are connected and the resulting curves will be referred to as the strobilation curves. These are estimates of the 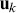 which are parameterized as a linear combination of cubic spline basis functions with maximum 10 degrees of freedom and further constrained to be a probability mass function. Recall that Table 1 indicates that Bristol Bay and St. Matthew Island were insignificant sources prior to 1990, so it is not meaningful to estimate their strobilation curves in the pre-1990 period. In general, the less productive a source, the more uncertainty in the strobilation curve estimation. Strobiliation timing for the Alaska Peninsula source was mainly in late March/early April. Later strobilation occurred at the Pribilof Islands (around late April/early May). For these two sources, there was no significant difference in strobilation timing between pre- and post-1990 periods, except for secondary release from the Alaska Peninsula in early June during the post-1990 period. In the post-1990 period, strobilation timing in Bristol Bay was in late March/early April while it was mainly April in St. Mathew Island.

We have carried out two experiments for assessing the impacts on the source-sink reconstruction of limitations in the circulation models ability to exactly reproduce actual ocean currents. Effects of the systematic negative bias in simulated current magnitudes were assessed by repeating the source-sink reconstruction using systematically shifted propagule data that correspond to increasing the simulated current magnitudes by a%, where a% ranges from 5% to 20% with a 5% increment. This range is chosen to represent typical underestimation of ocean velocity in ROMS simulations. Because of the extent of the 8 regions in the EBS, the covariates 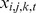 derived from the shifted ROMS data were only slightly perturbed from the original covariates. Consequently, the source-sink reconstruction results based on the shifted ROM outputs were also similar to the unperturbed model fit. In particular, the estimated sparsity linkage patterns for both pre- and post-1990 periods remained unchanged up to a shifting rate of 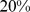 while the non-zero parameter estimates changed little, see Table S1. The estimated strobilation curves were generally robust to the shifting, with the shape of the curves mostly preserved (Fig. S8). Effects of random errors in the modelled ocean currents were assessed by refitting the model using perturbed 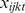
’s which were obtained by randomly adding or subtracting from each 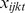 by 5% of its original value. The procedure was repeated 300 times. Model fits using the randomly perturbed datasets differed little from the fit based on the original data; the average misclassification rates, calculated from contrasting the estimated sparsity patterns from the perturbed datasets with those of the original data, are 4.36% and 4.28% for the pre-1990 period and post-1990 period, respectively. These results lend additional support to the robustness and validity of our modeling results on the jellyfish system.

## Discussion

[23] have postulated three hypotheses for the observed change in jellyfish distributions: (i) stronger oceanic transport from the southeastern EBS to the northwestern EBS carried more jellyfish to the northwest after 1990, (ii) warmer temperatures after 1990 allowed increased jellyfish production or allowed polyps to proliferate in the northwestern part of the survey area, and (iii) the combined effect of transport changes and increased productivity resulted in changes in jellyfish distribution. The source-sink models fitted to the pre- and post-1990 periods show both increased jellyfish productivity in all 4 sources and more extensive advection of jellyfish into new sinks in the post-1990 period than in the pre-1990 period, lending support to hypothesis (iii). Of significance is the fact that both Bristol Bay and St. Matthew Island changed from insignificant to significant sources in the post-1990 period, illustrating that potential sources may turn into productive sources with the onset of suitable environmental conditions.

These results rest on a number of assumptions that we had to make about the source-sink system. We assume that the observed adult jellyfish abundance in the eight sink locations are accounted for by the propagules generated within the four source locations (i.e., the 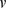
’s must sum to 1). This constraint generates some dependencies on model results and does not account for the fact that some of the simulated propagules may leave the system through the north, beyond region 8. However, particles that leave the system do not affect the results, because the imposed analytical constraint is contingent upon the observed adult jellyfish, which we assume to be originated from the four identified sources. We have identified four source locations in correspondence of hard and shallow substrate, which constitute preferred habitat features for benthic polyp colonies. There can be other regions within our modeled area that have these characteristics, especially along the Alaska Peninsula and in Bristol Bay (Fig. S5). Given the directionality of the shelf circulation (from southeast to northwest) it is unlikely that these additional sources can explain the increase of jellyfish in the central and northwestern area of the sampled region (areas 4 and 5). In fact, neither the Alaska Peninsula nor the Bristol Bay were significant sources for regions 4 and 5 (Table 1). Thus adding more source locations in the southeastern portion of the grid would not explain either the occurrence or the increase of central and northern jellyfish after 1990. We also have not considered possible source locations in the Pacific south of the Bering Sea. These potential sources could contribute to Bering Sea jellyfish populations via advection through the Aleutian passes but that analysis is beyond the scope of our analysis. We finally assume that the locations of the sink in a given year do not affect the source in the following year, because every year we release the same number of particles. Because polyp sources are constrained by depth and substrate, it is unlikely that the location of the adult jellyfish can substantially modify the location of the sources. However, the locations of the adult jellyfish can affect the productivity of the sources, by generating more larvae and settled polyps – an effect that is partly captured by the estimates of the 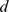 coefficients.

The estimated strobilation curves for the four sources reveal several interesting features. Along the Alaska Peninsula, jellyfish strobilation appeared to be trimodal, with significant strobilation throughout the 80-day window in the pre-1990 period. The strobilation pattern of this region, however, became essentially bi-modal in the post-1990 period, with the second peak in late May/early June. The strobilation curve suggests that there may be significant strobilation beyond early June, as observed in occasional plankton sampling in the area (Fig. S4). A different strobilation process, however, emerged in the Pribilof Islands, as the strobilation curve appeared to be identical in the pre- and post-1990 periods; the strobilation curve of the source there was unimodal with peak productivity around end of April. These findings are consistent with the hypothesis that a temperature cue is important in strobilation because Pribilof Islands is farther north and sometimes under the influence of heavy sea ice and thus warm later.

As the Alaska Peninsula was the most significant and productive source in both pre-1990 and post-1990 periods, a comparison of its two strobilation curves is very informative. (This region has been historically known as slime bank.) The mode of the strobilation curve estimate shifted to the right, suggesting later strobilation in the post-1990 period than the pre-1990 period. An interesting question naturally arises as to why, from the pre-1990 period to the post-1990 period, the source along the Alaska Peninsula both increased the intensity and altered the shape of the strobilation process, while the source around the Pribilof Islands only increased the strobilation intensity. Based on bottom temperature measurements from the trawl survey (after adjusting for variation of sampling days), the average summer bottom temperature over survey sites along the Alaska Peninsula increased from 2.88°C in the period prior to 1990 to 3.59°C in the post-1990 period. On the other hand, the bottom temperature around the Pribilof Islands increased from 3.12 to 3.35°C across the two periods. In the laboratory, scyphozoan strobilation is influenced by warming temperatures, i.e., longer strobilation periods and higher ephyra production [44]. Thus, the much larger increase in bottom temperature along the Alaska Peninsula may have triggered the more dramatic shape and intensity change in the strobilation pattern there. The strobilation curves for sources around Bristol Bay and St. Matthew Island are estimated only for the post-1990 period over which they are estimated to be significant sources. The source around Bristol Bay had a unimodal strobilation curve, with jellyfish productivity peaking in early April, and negligible production after late April. In contrast, the strobilation curve of the St. Matthew Island source was bimodal, with productivity starting later than the more southern sources. Note that, across all sources and the two periods of study, the lower limit of the individual 90% confidence intervals of the strobilation curves generally coincide with the x-axis, owing to the non-negativity constraint and the indirect nature of the information regarding strobilation.

The sensitivity analysis shows that our source-sink reconstruction for the jellyfish system in the EBS is robust to typical errors in the ROMS simulation of ocean circulation for the study area. In our approach, the EBS is subdivided into 8 relatively large contiguous sink regions. Were these sink regions more numerous, fractious and smaller in size, it would place greater demand on the ocean model accuracy to maintain the robustness of the results. Thus, the sensitivity analysis provides some support for the adopted 8-sink division scheme for the EBS.

Our statistical approach for source-sink dynamics reconstruction is developed for the case that field observations on the sinks are available but those in potential sources are scarce or even non-existent, while aggregate time-series data indicating the “strength” of contributions from any source to any sink are available. Such asymmetry on feasibility of taking observations in sinks but not sources arises in many applications, e.g. marine species with large knowledge gaps about their spawning process, or disease dynamics. Source-sink dynamics may, however, be studied via individual-based modeling accounting for the birth and death process, see [45]. Although the latter approach may yield more insights on the process, it requires observations from all sources and sinks, and knowledge on the dynamics of individuals, which are generally lacking in most applications.

Several future research directions include generalizing the proposed model to incorporate covariates, serially and/or contemporaneously correlated errors of unequal variance, which will extend the applicability of the proposed source-sink reconstruction framework. As well, it is of interest to investigate the theoretical properties of the (extended) approach.

## Supporting Information

Figure S1 Annual spatial distribution of jellyfish in four selected years, showing a northward shift in distribution after 1990. Sampling sites are depicted by dots. Isobath contours (50 m, 100 m, and 200 m) are shown (gray lines).
(TIF)

Figure S2 Locations of the sampling sites over the 8 sink regions.
(TIF)

Figure S3 Distribution of immature medusae (i.e., ephyrae) on the eastern Bering Sea shelf in summer of 1997–1998. Isobath contours (50 m, 100 m, and 200 m) are shown (gray lines). Samples were collected with a CalCOFI Vertical Egg Tow (CalVET) net and Multiple Opening/Closing Net and Environmental Sampling System (MOCNESS) by K. Coyle.
(TIF)

Figure S4 Day and depth of occurrence of scyphomedusan ephyrae in zooplankton tows collected on the eastern Bering Sea shelf. Samples were collected with a Multiple Opening/Closing Net and Environmental Sampling System (MOCNESS) on cruises in late spring (ca. 21 May–21 Jun) and late summer (ca. 22 Jul–11 Sep) in 1997–1999 by K. Coyle.
(TIF)

Figure S5 Distribution of bottom types in the EBS.
(TIF)

Figure S6 Normal Q-Q plots. Left: pre-1990 model; right: post-1990 model.
(TIF)

Figure S7 Left: residuals vs. fitted values; right: observed values vs. fitted values.
(TIF)

Figure S8Release time (strobilation) curves (the estimated 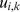
’s) for the four potential sources based on “shifted” ROM outputs for the periods 1982–1990 (upper 2 panels) and 1991–2004 (lower 2 panels), with the rate of shifting 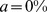 to 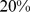, with increments 5%. During the pre-1990 period, the release time curve estimates based on the shifted data are almost identical and not visibly distinguishable for each of the sources, except Alaska Peninsula.(TIF)

Table S1Sensitivity analysis: model fitting results based on “shifted” ROM outputs, with the rate of shifting 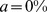 to 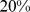, with increments 5%. Since the age patterns estimated from the shifted data are identical to that based on the original data, we only report the nonzero parameter estimates.(PDF)

File S1 Contains a link to download the covariate data, further details on the residual bootstrap, and model fitting and diagnostics.
(PDF)
