# Supplementary material for: Reconstructing Source-Sink Dynamics in a Population with a Pelagic Dispersal Phase
Source: PLoS One. 2014 May 16;9(5):e95316. doi: 10.1371/journal.pone.0095316 (PMC4023943; doi:10.1371/journal.pone.0095316)
Supplement: Table S1 — Sensitivity analysis: model fitting results based on “shifted” ROM outputs, with the rate of shifting to , with increments 5%. Since the age patterns estimated from the shifted data are identical to that based on the original data, we only report the nonzero parameter estimates. (PDF) [file pone.0095316.s009.pdf]

Table S1. Sensitivity analysis: model fitting results based on ''shifted" ROM outputs, with the rate of shifting $a = 0\%$ to 20%, with increments 5%. Since the age patterns estimated from the shifted data are identical to that based on the original data, we only report the nonzero parameter estimates.

			Pre-1990 Period		
	Original	5%	10%	15%	20%
m_1	1.650	1.617	1.610	1.624	1.617
m_2	0.160	0.161	0.161	0.162	0.163
m_3	0.496	0.496	0.497	0.498	0.497
m_4	0.156	0.157	0.157	0.156	0.156
$v_{3,1}$	0.070	0.056	0.053	0.066	0.066
$v_{6,1}$	0.930	0.944	0.947	0.934	0.934
$v_{1,2}$	1.000	1.000	1.000	1.000	1.000
$v_{4,3}$	0.079	0.079	0.080	0.080	0.080
$v_{7,3}$	0.921	0.921	0.920	0.920	0.920
$v_{5,4}$	1.000	1.000	1.000	1.000	1.000
			Post-1990 Period		
	Original	5%	10%	15%	20%
m_1	2.764	2.790	2.778	2.781	2.865
m_2	0.625	0.624	0.625	0.626	0.626
m_3	1.784	1.853	1.990	1.941	1.769
m_4	1.715	1.666	1.631	1.614	1.557
$v_{3,1}$	0.110	0.107	0.110	0.109	0.096
$v_{4,1}$	0.411	0.396	0.370	0.353	0.432
$v_{6,1}$	0.479	0.497	0.520	0.537	0.472
$v_{1,2}$	1.000	1.000	1.000	1.000	1.000
$v_{4,3}$	0.006	0.006	0.004	0.005	0.014
$v_{5,3}$	0.007	0.008	0.006	0.008	0.019
$v_{7,3}$	0.048	0.052	0.035	0.044	0.132
$v_{8,3}$	0.939	0.933	0.955	0.943	0.836
$v_{5,4}$	0.014	0.015	0.015	0.018	0.022
$v_{8,4}$	0.986	0.985	0.985	0.982	0.978
