# Supplementary material for: Reconstructing Source-Sink Dynamics in a Population with a Pelagic Dispersal Phase
Source: PLoS One. 2014 May 16;9(5):e95316. doi: 10.1371/journal.pone.0095316 (PMC4023943; doi:10.1371/journal.pone.0095316)
Supplement: File S1 — Contains a link to download the covariate data, further details on the residual bootstrap, and model fitting and diagnostics. (PDF) [file pone.0095316.s010.pdf]

Reconstructing Source-Sink Dynamics in a Population with a Pelagic Dispersal Phase
Supplemental File

The Covariate Data

The covariate data, i.e. the x's, used in the jellyfish source-sink reconstructions can be downloaded at http://homepage.stat.uiowa.edu/~kchan/source-sink.htm

Bootstrap

Define the set of centered residuals $\{\check{e}_i = \hat{e}_i - \frac{1}{Tq}\sum_{j=1}^{Tq}\hat{e}_j; i = 1, ..., Tq\}$. We randomly select Tq error terms with replacement, $\mathbf{e}^{+} = (e_1^{+}, ..., e_{Tq}^{+})$, from the set of centered residuals and formulate the bootstrap sample $\mathbf{y}^{+} = \hat{\mathbf{y}} + \mathbf{e}^{+}$. The bootstrap estimates of the parameters are then found by fitting the bootstrap data using the proposed penalized estimation method. This process is repeated a number of times (400 times in the numerical work herein) to obtain the bootstrap distributions of the parameters of interest.

Model Fitting and Diagnostics

The optimization of the objective function is conducted by an iterative algorithm which alternately updates the parameters source by source until convergence, as follows. Suppose we want to update all the parameters related to the hth source. Let $y_{j,t}^{(h)} = y_{j,t} - \sum_{k\neq h}^{K}(d_k v_{j,k}\sum_{i=1}^{p} u_{i,k}x_{i,j,k,t})$. Upon fixing all parameters except those of the hth source, the optimization problem becomes minimizing $\sum_{j,t}\{y_{j,t}^{(h)} - d_h v_{j,h}\sum_{i=1}^{p} u_{i,h}x_{i,j,h,t}\}^2 + \lambda_h \sum_{i=1}^{p}\sum_{j=1}^{q} w_{i,j,h}|d_h u_{i,h} v_{j,h}|$ with respect to $(d_h, u_{i,h}, v_{j,h})$, $i = 1, \ldots, p$, $j = 1, \ldots, q$. This much simpler problem can be efficiently solved by an alternating lasso method; see [1] for details. We use AIC to determine the optimal value of λ_h. Denote the optimizer as $(\hat{d}_h^{(\lambda_h)}, \hat{u}_{i,h}^{(\lambda_h)}, \hat{v}_{j,h}^{(\lambda_h)})$, with the tuning parameter being λ_h. Define

$$\mathrm{AIC}(\lambda_h) = \log(\mathrm{SSE}(\lambda_h)) + \frac{2}{Tq}df(\lambda_h),$$

where $\mathrm{SSE}(\lambda_h) = \sum_{j,t}\{y_{j,t}^{(h)} - \hat{d}_h^{(\lambda_h)}\hat{v}_{j,h}^{(\lambda_h)}\sum_{i=1}^{p}\hat{u}_{i,h}^{(\lambda_h)}x_{i,j,h,t}\}^2$. Following [2], the degrees of freedom is given by

$$df(\lambda_h) = \sum_{i=1}^{p}\mathrm{I}(\hat{u}_{i,h}^{(\lambda_h)} \neq 0) + \sum_{j=1}^{q}\mathrm{I}(\hat{v}_{j,h}^{(\lambda_h)} \neq 0) - 1,$$

where $\mathrm{I}(\cdot)$ is the indicator function. We compute the solutions over a grid of 100 equally spaced λ_h values on the log scale, between $\lambda_{\min} = 0$ and $\lambda_{\max}$, the smallest λ_h value at which all coefficients become zero [1]. The best λ_h is selected as the one yielding the smallest AIC. Following [2], the selection of the K regularization parameters is nested within the iterative algorithm, in order to avoid the computationally expensive high-dimensional grid search. For the fitted pre-1990 model, the optimal tuning parameters are 0.010, 0.018, 0.003 and 0.013 ($\times 10^{-5}$), for Alaska Peninsula, Bristol Bay, Pribilof Islands and St. Matthew Island, respectively; their post-1990 model counterparts are 0.015, 0.041, 0.001 and 0.001 ($\times 10^{-5}$). While these λ values may seem small in magnitude, the degree of penalization is also determined by the adaptive weights $w_{i,j,k} = (\tilde{d}_k\tilde{u}_{i,k}\tilde{v}_{j,k})^{-2}$ where $(\tilde{d}_k, \tilde{u}_{i,k}, \tilde{v}_{j,k})$ are the least squares estimators [3]. Besides, the enforced nonnegativity constraints also promote sparsity in the estimates of

the u's and v's, thereby lessening the penalty needed for recovering certain sparsity pattern.

The variability in the observed jellyfish CPUE data $y_{j,t}$ is substantial, which reflects the complexity and inhomogeneity of the spatial/temporal dynamics of the distribution of jellyfish. Most of the CPUE observations in the data are small, with the 50th and 75th percentiles being 0.57 and 2.03, respectively; a few observations are quite large, with the 95th percentile and the maximum being 7.26 and 22.9, respectively, indicating occasional large fluctuations in the jellyfish biomass. To alleviate the influence/domination of the few large observations, the natural log-transformed CPUE, $log(y_{j,t}+1)$, is found to fit the proposed source-sink model well, i.e. $\log(y_{j,t}+1) = f_{j,t} + e_{j,t}$ where $f_{j,t} = \sum_{k=1}^{K}\{d_k v_{j,k} \sum_{i=1}^{p} u_{i,k} x_{i,j,k,t}\}$. Since $\log(y_{j,t}+1) \approx y_{j,t}$ when $y_{j,t}$ is small, this transformation has little effect on the majority of the observations in the data. In fact, the linearity of the model remains to hold approximately. To see this, let f_j be the expected value of $f_{j,t}$. By Taylor expansion, $y_{j,t} \approx (f_{j,t} - f_j + 1)\exp(e_{j,t} + f_j)$ when $f_{j,t} - f_j$ is small, which means that $y_{j,t}$ is approximately linearly related to $f_{j,t}$. Therefore, the model interpretations in the source-sink reconstruction with the log-transformed data remain valid. For future research, it would be worthwhile to consider a more complex model with the original data, by incorporating a thick-tail distribution that accounts for the occasional large observations.

We have evaluated the goodness of fit of the fitted source-sink reconstruction model, by checking whether the errors satisfy the independent and identical distribution (i.i.d.) assumption. The residuals from the pre-1990 and post-1990 periods are combined and standardized region by region. The autocorrelation patterns of the residuals from each region have been checked, and no significant autocorrelation is found up to lag 12 for each residual series. The cross correlation patterns between each pair of prewhitened regional residual series (28 pairs) have also been examined. There are only 4 significant cross correlations in all pairs of residuals up to lag 6 (13 cross correlations per each pair), i.e. the number of significant lags is about 1%. Hence, the standardized residuals appear to satisfy the i.i.d. assumption.

We have also checked the normality assumption with the residuals. Fig. S6 displays the Normal Q-Q plots for the combined residuals, and no clear departure form normality can be seen. Based on the Shapiro-Wilk normality test, the normality of each set of residuals are not rejected at the 5% significance level. We note that our proposed method does not require the error term to be normally distributed. Fig. S7 displays (1) the residuals vs. the fitted values and (2) the observed values vs. the fitted values. Our model appears to fit the data well, and except for a few possible outliers, the residuals do not show any discernible pattern.

We use a permutation approach to further assess the model validity, especially to assess the significance of the structural change in the source-sink dynamics starting in 1990. Under the null hypothesis of no change in the source-sink dynamics, we can shuffle the datacases by permuting the year. For each permutated data, we refit the model to the two separate periods (pre- and post-1990) and compute a test statistic defined as the sum of the AIC of the model using data from the "pre-1990" period and the AIC of that from the "post-1990" period. This procedure is repeated 500 times, and a reference distribution is built for the test statistic. The observed test statistic using the actual data is 4.56, and it is smaller than all the 500 values based on permuted data, i.e., the estimated p-value of no change, computed as the fraction of the 500 values based on permutated datasets being less than the observed value 4.56 based on true data, is < 0.002. This result provides strong evidence that a change occurred in the source-sink dynamics starting 1990.

Altogether, we can conclude from these model diagnostics that our fitted model is correctly specified and provides a good fit to the data.

References

1. Chen, Kun and Chan, Kung-Sik and Stenseth, Nils Chr. (2014) Source-sink Reconstruction through Regularized Multi-component Regression Analysis – With Application to Assessing Whether North

Sea Cod Larvae Contributed to Local Fjord Cod in Skagerrak. To appear in Journal of the American Statistical Association.

2. Chen, K and Chan, K.-S. and Stenseth, N. C. (2012) Reduced rank stochastic regression with a sparse singular value decomposition. Journal of the Royal Statistical Society: Series B (Statistical Methodology) 74: 203–221.

3. Zou, Hui (2006) The adaptive lasso and its oracle properties. Journal of the American Statistical Association 101: 1418–1429.
